# Glucosinolates and Polyphenols of Colored Cauliflower as Chemical Discriminants Based on Cooking Procedures

**DOI:** 10.3390/foods11193041

**Published:** 2022-09-30

**Authors:** Ancuta Nartea, Benedetta Fanesi, Alessandra Giardinieri, Guillem Campmajó, Paolo Lucci, Javier Saurina, Deborah Pacetti, Dennis Fiorini, Natale Giuseppe Frega, Oscar Núñez

**Affiliations:** 1 Department of Agricultural, Food and Environmental Sciences, Marche Polytechnic University, Via Brecce Bianche, 60131 Ancona, Italy; 2 Department of Chemical Engineering and Analytical Chemistry, University of Barcelona, Martí i Franquès 1-11, 08028 Barcelona, Spain; 3 Research Institute in Food Nutrition and Food Safety, University of Barcelona, Av. Prat de la Riba 171, Edifici Recerca (Gaudí), 08901 Santa Coloma de Gramenet, Spain; 4 Chemistry Division, School of Science and Technology, University of Camerino, V. S. Agostino 1, Camerino, 62032 Macerata, Italy

**Keywords:** bioactive compounds, boiling, cauliflower, cruciferous, glucosinolates, phenolics, steaming, *sous-vide*

## Abstract

The impact of mild oven treatments (steaming or *sous-vide*) and boiling for 10 min, 25 min, or 40 min on health-promoting phytochemicals in orange and violet cauliflower (*Brassica oleracea* L. var. botrytis) was investigated. For this purpose, targeted ultra-high performance liquid chromatography–high-resolution mass spectrometry analysis of phenolics and glycosylates, combined with chemometrics, was employed. Regardless of cooking time, clear differentiation of cooked samples obtained using different procedures was achieved, thus demonstrating the distinct impact of cooking approaches on sample phytochemical profile (both, compound distribution and content). The main responsible components for the observed discrimination were derivatives of hydroxycinnamic acid and kaempferol, organic acids, indolic, and aromatic glucosinolates, with glucosativin that was found, for the first time, as a discriminant chemical descriptor in colored cauliflower submitted to steaming and *sous-vide*. The obtained findings also highlighted a strict relationship between the impact of the cooking technique used and the type of cauliflower. The boiling process significantly affected the phytochemicals in violet cauliflower whereas orange cauliflower boiled samples were grouped between raw and either steamed or *sous-vide*-cooked samples. Finally, the results confirm that the proposed methodology is capable of discriminating cauliflower samples based on their phytochemical profiles and identifying the cooking procedure able to preserve bioactive constituents.

## 1. Introduction

In the last couple of decades, vegetables from the genus Brassica, commonly known as cruciferous vegetables, have received great attention due to the presence of secondary metabolites. Brassicaceae vegetables are vital dietary sources of phenolic compounds [1], flavonoids, and hydroxycinnamic acid glycosides derivatives are the most abundant [2]. These compounds constitute a wide group of phytochemicals providing health benefits due to their antioxidant [3,4] and anti-inflammatory activity [5]. Moreover, they are employed in the treatment of obesity [6], type 2 diabetes [7], metabolic syndrome [8], neurodegenerative diseases [9,10], atherosclerosis [11], and cancer [12,13,14,15].

The Brassicaceae family is also characterized by the presence of glucosinolates [13,16]. Many studies have been carried out about glucosinolates and their enzymatically hydrolyzed products (isothiocyanates) for their health-promoting properties and therapeutic benefits [17] such as anti-cancerogenic [18,19,20,21,22], anti-inflammatory [23], as well as anti-diabetogenic [24,25] effects.

Lately, cauliflower (*Brassica oleracea* L. var. botrytis) has received increasing attention due to its varietal proximity to broccoli, one of the most widely investigated cruciferous species [26,27,28,29]. Colored cauliflowers, such as orange and violet, have enjoyed a commercial resurgence. Such vegetables exhibit diversification in both their chemical composition and, consequently, the level of bioactive chemicals they possess [13,30].

Considering that cauliflowers are usually heat-treated before consumption, particular attention needs to be given to the cooking steps employed [26], as these could influence the profile and content of bioactive compounds due to two contrary phenomena. The first one is the degradation of bioactive compounds caused by different mechanisms. The other phenomenon is the ability to release these substances from the food material, which can be altered during cooking, boosting some bioactive chemicals’ bioaccessibility [30]. The overall impact of cooking on phytochemical content, therefore, relies on the processing parameters, the form of the vegetable tissue, and the chemical makeup of the compound [31,32].

To the best of our knowledge, very few studies have investigated changes in the polyphenols or glucosinolates profiles [33,34,35]. The majority of the studies have centered on the effect of cooking on the total level of polyphenols or glucosinolates in cauliflowers with high variability of data [36]. For polyphenols in white cauliflower, dos Reis et al. [37] found that boiling, steaming, and *sous-vide* procedures caused a reduction in the total phenolic content, while other authors have revealed that steaming preserved the phenolic compounds [38,39]. In violet cauliflower, the negative impact of boiling on the total polyphenols was higher when compared to the white cauliflower literature [30]. For glucosinolates in white cauliflower, steaming had a favorable impact on their content, limiting the losses [40,41]. Contents of hydrolyzed glucosinolate compounds present in genus *Brassica* also decreased significantly with boiling treatments, with a reduction of 11% and 42.4% for green and purple cooked cauliflower, respectively [34].

To give an overview of the impact of different domestic cooking methods on cauliflower bioactive compounds, glucosinolates and polyphenols profiles were used as chemical descriptors to discriminate between cooking procedures. Cheddar (orange) and Depurple (violet), two newly colored varieties of cauliflower (*Brassica oleracea* L. var. botrytis) were selected. Mild thermal oven treatments with rapid heating using steam as heat transfer medium (steam and *sous-vide* oven) were tested at different cooking times and compared with a water boiling cooking method. A targeted ultra-high performance liquid chromatography–high-resolution mass spectrometry (UHPLC–HRMS) method was employed to obtain the cauliflower phytochemical profiles using accurate mass databases, including phenolic and glucosinolate compounds. Glucosinolates and polyphenols profiles were then employed as chemical descriptors to address sample discrimination based on cooking procedures and cooking time, employing non-supervised (principal component analysis, PCA) and supervised (partial least squares regression-discriminant analysis, PLS-DA) chemometric methods. A detailed study of PLS-DA using variable importance in projection (VIPs) allowed a further selection of the most discriminant phytochemicals among the targeted phenolics and glucosinolates to characterize samples according to the sample cooking procedures and cooking time.

## 2. Materials and Methods

### 2.1. Reagents and Materials

LC-MS grade water, methanol (MeOH), acetonitrile (ACN), acetone for pesticide residue analysis (purity ≥ 99.8%), as well as formic acid (≥98%) from Sigma-Aldrich (Steinheim, Germany), were employed. Nitrogen (>99%), used for the quadrupole-Orbitrap system, was provided by Linde (Barcelona, Spain).

### 2.2. Instrumentation

Regarding the chromatographic separation, an Accela UHPLC system (Thermo Fisher Scientific, San José, CA, USA), equipped with a quaternary pump and an autosampler, was employed. Following a previously published procedure, the separation was performed using an Ascentis Express C18 column (150 2.1 mm, 2.7 m partly porous particle size). The mobile phase consists of water as solvent A and acetonitrile as solvent B, both acidified with 0.1% formic acid (*v*/*v*) [42]. The gradient elution program used was: 0–1 min, isocratic conditions at 10% B; 1–20 min, linear gradient until 95% B; 20–23 min, isocratic step at the previous composition; and from 23 to 30 min, back to initial conditions and re-equilibration of the column. The injection volume used (in filled loop capacity) was 10 µL, while the chromatographic column was at room temperature.

On the other hand, concerning the mass spectrometric acquisition, a Q-Exactive Orbitrap HRMS (Thermo Fisher Scientific, San José, CA, USA), which was coupled to the UHPLC system by using a heated-electrospray ionization source (HESI-II) working in negative mode, was employed. Nitrogen was used for the H-ESI sheath, ion sweep, and supporting gases at flow rates of 60, 0, and 10 a.u. (arbitrary units), accordingly. Moreover, a capillary voltage of −2.5 kV, vaporizer temperature of 350 °C, ion transfer tube temperature of 320 °C, and an S-Lens RF level of 50%, were set. The Q-Exactive Orbitrap system, which was tuned and calibrated for both positive and negative modes every 3 days using commercially available Thermo Fisher calibration solutions, was operated in negative full MS mode (*m*/*z* 100–1500) at a mass resolution of 70,000 full widths at half-maximum (FWHM) at *m*/*z* 200, with an automatic gain control (AGC) target of 1.0 × 10^6^ and maximum injection time of 200 ms.

For the control of the entire LC-MS system as well as data acquisition and processing, Xcalibur software version 2.1 was used (Thermo Fisher Scientific, San José, CA, USA).

### 2.3. Samples and Sample Treatment

Fresh violet (Depurple cv.) and orange (Cheddar cv.) cauliflowers were cultivated and purchased from the local producer AGRINOVANA s.r.l. of Petritoli (Fermo, Italy). Vegetable samples were directly prepared for analyses after harvest. Before thermal processing, the initial step of processing was removing the leaves and any broken portions, washing them under flowing water, and splitting the heads into roses that were 3–4 cm in diameter and 4–5 cm in length, and weighed 9–12 g. Cutting the cauliflower required the use of a stainless-steel knife. To create the representative average laboratory samples, the vegetables were combined, and then divided into 300 g parts for the cooking process. Fresh vegetables were subjected to analyses immediately following preparation.

Cauliflowers were boiled in 1.5 L of unsalted water at the boiling temperature in a steel pot (height = 21 cm; diameter = 18 cm) for 10 and 25 min. Steam was injected into a chamber (RH% = 100) of an oven (Bosch, Germany,) obtained from a regional distributor (Media World Italy) to cook the vegetable at the temperature of 95 °C (for 10, 25, 40 min). The cauliflowers were steamed as described above for *sous-vide* (SV) oven cooking before being vacuum-packed in a polypropylene heat-resistant (up to 120 °C) bag. Every cooking experiment was carried out thrice. The parameters of the cooking conditions were chosen to mimic general consumer habits. Thus, 40 min of boiling was excluded as it turns the sample into a mush. After cooking, cauliflowers were removed from the heat, allowed to cool at ambient temperature, then cut into small pieces with a knife, freeze-dried using a Virtualis Wizard 2.0 device from SP Industries in New York, ground, and then kept in vacuum bags at −18 °C [31].

The sample extraction procedure was carried out following a previously documented procedure, with a few adjustments [42]: 0.3 g of lyophilized vegetable sample was extracted with 3 mL of acetone:water:formic acid (70:29.9:0.1 *v*/*v*/*v*) solution by sonication for 15 min, and then vortexed for 1 min. The supernatant extracts were obtained after centrifugation (3400 rpm, 15 min) and filtration (0.45 μm nylon filters, Whatman, Clifton, NJ, USA), and they were stored at −4 °C until the UHPLC–HRMS analysis.

Additionally, a quality control (QC) sample was made by combining 75 mL of each sample extract to examine the repeatability of the suggested approach and to analyze the robustness of the chemometric data. Random UHPLC-HRMS analyses were performed and for every ten samples, QCs and acetonitrile blanks were examined.

### 2.4. Data Analysis

UHPLC–HRMS raw data were processed using TraceFinderTM software (Thermo Fisher Scientific, San José, CA, USA), by the application of a user target mass database list comprising several phenolic and glucosinolate compounds. Moreover, different parameters such as the peak intensity threshold of 5 × 10^5^, mass measurement error below 5 ppm, and isotope pattern fit with values higher than 85%, were established for recognition and confirmation purposes. Then, a feature matrix, with each molecular feature represented by its *m*/*z* and retention time, was built and subjected to chemometric methods. The MS/MS experiments and fragmentation pattern comparison were not performed.

The PCA and PLS-DA study used the SOLO 8.6 chemometric program from Eigenvector Research (Manson, WA, USA) (http://www.eigenvector.com/software/solo.htm; accessed on 19 January 2021). While PCA was only used to check the correct behavior of the chromatographic results through QCs samples, PLS-DA was used as a supervised classificatory method to observe the discrimination of samples according to their cooking procedure and time by differences in their phytochemical profile. [43]. Data were autoscaled to equalize the influence of each variable in the model, using the average (x $\overset{-}{x}$) and the standard deviation (s) of each variable as follows: (xi − $\overset{-}{x}$)/s, with xi being the original data. Moreover, the highest VIP values were used to detect the most discriminant molecular features.

## 3. Results and Discussion

### 3.1. UHPLC–HRMS Phytochemical Profiling

**Figure 1:**
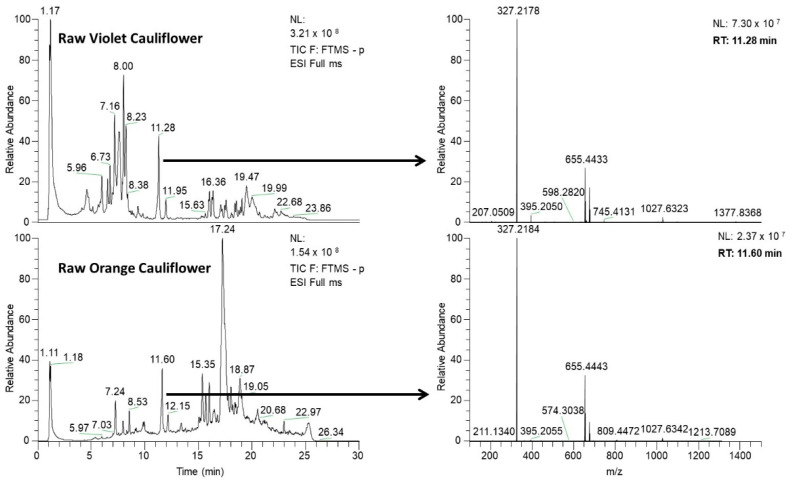
UHPLC–HRMS fingerprints (total ion chromatogram, TIC) for the raw violet and orange cauliflower samples. Full HRMS spectra for each sample fingerprint at a given chromatographic retention time are also depicted.

In the present contribution, changes in the phytochemical profiles of both violet and orange cauliflowers processed by different cooking procedures (boiling, steaming, and *sous-vide*) performed at different cooking times (10, 20, and 40 min) were studied. As an illustration, Figure 1 shows the obtained UHPLC–HRMS chromatograms (total ion chromatogram, TIC) for both raw violet and orange cauliflower samples, as well as the full HRMS spectra for each sample at a given chromatographic retention time.

As can be seen, there are noteworthy differences in the acquired chromatograms (distribution and abundance of signals) that may be related to phenolic and glucosinolate content variations. Therefore, the obtained HRMS spectral data were processed by using an accurate mass database of specific phytochemicals using TraceFinderTM software. This accurate mass database included a total of 61 phytochemical compounds (see Table S1) belonging to organic acids, phenolics (i.e., hydroxybenzoic acids, derivatives of hydroxycinnamic acids, isorhamnetin, kaempferol, and quercetin) and glucosinolates (aliphatic, indolic, and aromatic) compounds. To obtain the cauliflower phytochemical profiles, several conditions were established in the TraceFinderTM screening program to consider the possibility that a component could tentatively exist in the analyzed sample (see Section 2.4). After raw data processing of the UHPLC–HRMS chromatograms, a report for each sample extract was obtained with those screened compounds found and selected according to the abovementioned criteria. It should be noted that TraceFinderTM does not differentiate among isobaric compounds (a match is given when an *m*/*z* value is found within the compounds included in the accurate mass database employed following the established screening criteria). Additionally, it is possible to monitor the presence of the same phytochemical (predicted *m*/*z* value) at several chromatographic periods within the same studied sample. This can frequently happen when the sample contains the native phytochemical as well as some of its derivatives (i.e., glycosylated derivatives or other adducts). In those cases, both compounds (native and derivative) are chromatographically well separated, but if the derivative compound suffers in-source collision-induced dissociation (CID) fragmentation when reaching the ESI source, it also yields the ion corresponding to the native phytochemical, and therefore, a match by TraceFinderTM software at different retention times for the native phytochemical will be given.

Finally, the obtained UHPLC–HRMS phytochemical profiles (peak areas) were employed as chemical descriptors for sample characterization and discrimination by chemometrics.

### 3.2. Phytochemical Profiles as Chemical Descriptors for Sample Discrimination

As a first approach, the UHPLC–HRMS phytochemical profiles for both violet and orange cauliflower groups of samples, together with the corresponding QCs, were submitted to an exploratory PCA model to evaluate the repeatability and robustness of the chemometric results. For that purpose, phytochemical profiles, defined as the peak area of those compounds found by TraceFinderTM screening software as a function of *m*/*z* ratio and retention time (if the same *m*/*z* ratio was detected at different retention times as previously commented), were employed as sample chemical descriptors. As an example, Figure S1 shows the PCA scores plot of PC1 versus PC2 for the analyzed orange and violet cauliflower samples. QCs appeared perfectly grouped close to the center of the plot despite the distribution of the analyzed samples, showing the good performance and reproducibility of the proposed methodology.

Once the good performance of the proposed UHPLC–HRMS methodology and phytochemical profiles were established, sample classification according to the cooking procedure and time was studied by a supervised classificatory method such as PLS-DA.

**Figure 2:**
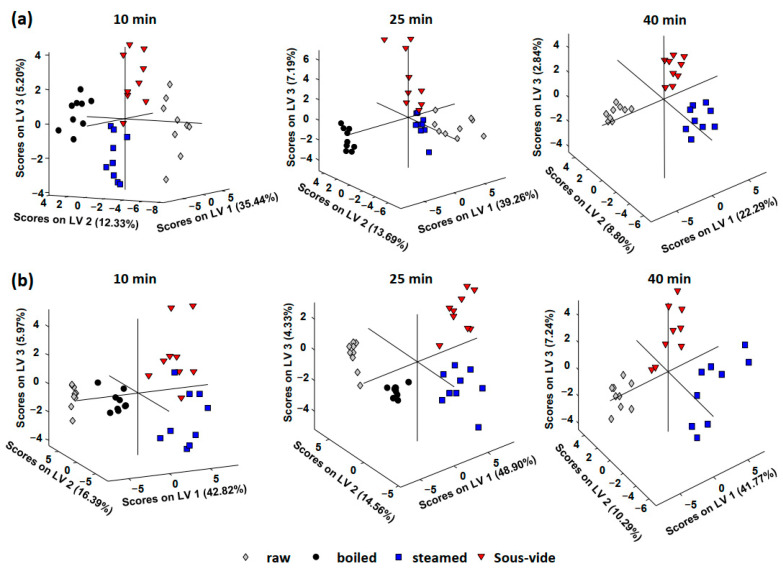
PLS-DA scores plots obtained using UHPLC–HRMS phytochemical profiles as chemical descriptors for the classification of (**a**) violet and (**b**) orange cauliflower samples according to the cooking procedure (raw samples, boiling, steamed, and *sous-vide*).

Figure 2 shows the PLS-DA scores plots obtained when the UHPLC–HRMS phytochemical profiles were employed as chemical descriptors for the classification of (a) violet and (b) orange cauliflower samples according to the cooking procedure (raw, boiled, steamed, and *sous-vide*-treated samples).

As can be seen in the plots, satisfactory results for both violet and orange cauliflower groups of samples were achieved. Independently of the cooking time used (10, 25, or 40 min), samples appeared perfectly grouped and differentiated according to the cooking procedure used, as well as not-cooked (raw) samples. This demonstrates that the sample phytochemical profile (both, compound distribution and content) is influenced by the cooking method employed and, therefore, the proposed UHPLC–HRMS phytochemical profiles are good sample chemical descriptors to guarantee cauliflower cooking procedure when necessary. From the obtained results, it can also be deduced that the effects caused by the cooking procedure employed are different depending on the type of cauliflower. Thus, in the case of violet cauliflower (Figure 2a), boiled samples are clustered more separately from the non-processed samples, while the opposite behavior was observed for the orange cauliflower, the boiled samples being grouped between the non-processed samples and those that were either steamed or *sous vide*-cooked. Specific changes in the phytochemical contents will be discussed in the next section.

**Figure 3:**
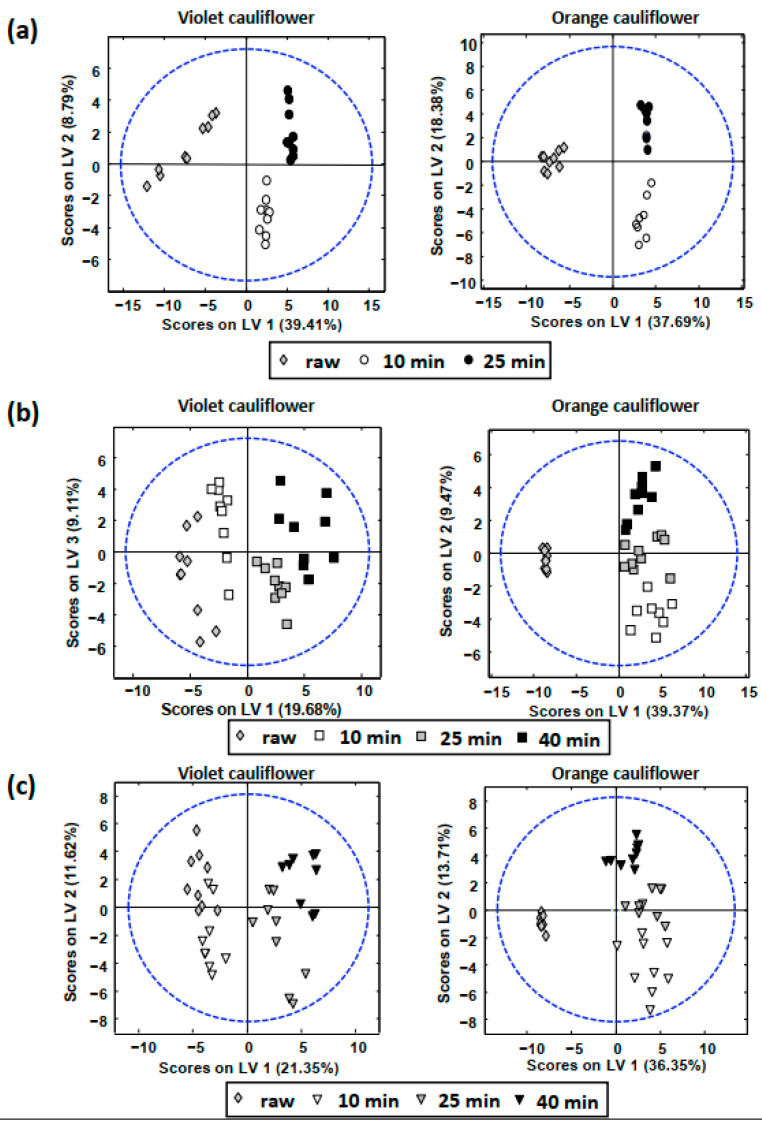
PLS-DA scores plots obtained using UHPLC–HRMS phytochemical as chemical descriptors to evaluate the classification of (**a**) boiled, (**b**) steamed, and (**c**) *sous-vide* samples, according to the cooking time employed.

UHPLC–HRMS phytochemical profiles were also employed as chemical descriptors to evaluate the classification of samples according to the cooking time employed, independently of the cooking method. Figure 3 shows the obtained PLS-DA models for (a) boiled, (b) steamed, and (c) *sous-vide* cauliflower samples.

As can be seen, samples tend to be grouped and distributed in different areas of the PLS-DA scores plots according to the cooking time, independently of the cooking procedure employed. These results show that the proposed UHPLC–HRMS phytochemical profiles are also good chemical descriptors to address cauliflower sample classification regarding the cooking time, demonstrating that changes in the phytochemical profile and contents are obtained, as expected, when increasing the cooking time. An important difference between violet and orange cauliflower sample behavior, especially when steaming and *sous-vide* cooking were employed (Figure 3b,c, respectively), is that higher cooking times are required for violet cauliflowers to observe important differences in the phytochemical profiles. This can be noticed by the fact that violet raw and 10-min cooked samples appeared clustered together, in the same area of the plots, in comparison to the case of orange cauliflowers, at least for steaming and *sous-vide* procedures. Nartea et al. [31] also showed that boiling, steaming, and *sous-vide* led to an increase in carotenoids and tocopherols extractability in cauliflowers. However, the magnitude of the rise varied depending on the type of cauliflower, the cooking conditions (temperature and duration), and the chemical makeup of the compound. In either scenario, boiling demonstrated a higher ability than steaming and sous viding to release all liposoluble antioxidants (carotenoids and tocopherols) from the cauliflower’s tissue when the cooking conditions were identical. Even though the *sous-vide* method affected more types of cauliflower than the steam oven, the latter treatment showed similar results. In actuality, the *sous-vide* cooking method proved successful in improving tocopherol in orange but not in violet cauliflower.

Once it was demonstrated that the phytochemical profiles of the analyzed samples were considerably modified according to the cooking procedures and the cooking time, a study of the loadings plots was carried out to identify which variables (phytochemical signals found in the samples using TraceFinderTM software) were responsible for the observed discrimination. For that purpose, those variables with the highest VIP values in the PLS-DA model of interest were revealed. The observed changes in the cauliflower phytochemical contents will be discussed in the next section.

### 3.3. Qualitative Changes in Phytochemical Contents

Based on the VIP study previously commented on, the signal level of the compounds selected as more discriminant for the sample classifications previously discussed was revealed (normalized signal with respect to the median of QCs) to observe the most important changes in the cauliflower phytochemical contents according to the cooking procedure and the cooking time. It should be mentioned that these are not quantitative results because of the lack of standards for all these chemicals. However, for each phytochemical, qualitative results regarding the increase or the decrease of its level in the analyzed samples can be extracted from the obtained data, although no comparison between phytochemical levels can be performed because the response factors are compound-dependent.

#### 3.3.1. Violet Cauliflower

The most discriminant phytochemical compounds for boiling were glucobrassicin and the derivatives of citric acid, hydroxycinnamic acid, and kaempferol. As an example, their variation level because of boiling treatment is summarized in Figure S2a. Three trends can be observed. The levels of glucobrassicin, citric and metilcitric acids, feruloylglucoside, and kaempferol-3,7-di-O-glucoside decreased after boiling. The pattern was quite the contrary for kaempferol 7-O-glucoside-3-O-acyl glucosyls and sinapic acid where a clear increase in their concentration level by boiling was observed, being enhanced with the boiling time. Finally, kaempferol-3-O-sinapoyl-sophorotrioside-7-O-glucoside and kaempferol-3-O-p-coumaroyl-sophoroside-7-O-diglucoside showed an increase in their concentration level while the samples were boiled, but then at higher boiling times the concentration decreases again almost reaching the same concentration level than the raw material after boiling for 25 min.

The release of sinapic acid could derive from the degradation and breakdown of more complex polyphenols. The same consideration was found for *p*-coumaric, ferulic, sinapic, gallic, and protocatechuic acids [44]. Acylation more than sugar moiety could explain the antagonistic behavior of kaempferol-3,7-di-O-glucoside (non-acylated) and kaempferol 7-O-glucoside-3-O-acyl glucosyls (acylated). Wu et al. suggested that acylation may provide heat resistance to compounds, as seven acylated tri or tetra-glycosides found in boiled broccoli showed fewer losses than kaempferol-3,7-di-O-glucoside [45]. Vegetable tissue softening is another key factor to explain compound levels. Cell disruption could have free and solubilized matrix-bounded phenolics [44].

Unlike boiling, all the levels of phytochemicals were found to be discriminants enhanced after steaming or *sous-vide* treatments (Figure S2b,c). The same VIP was revealed for both treatments. In detail, a constant increase in concentration level with steaming and *sous-vide* time was obtained for derivatives of hydroxycinnamic acids (i.e., caffeoyl-quinic acid, courmaroyl-diglucoside, and sinapyl-glucoside) and kaempferol (kaempferol 7-O-glucoside-3-O-acyl glucosyls and kaempferol 7-O-glucoside). Differently, a 10-min treatment is enough (steaming or *sous-vide*) to obtain the highest level of glucosativin (4-mercaptobutylglucosinolate).

Analyzing the level variation of the most discriminant phytochemicals obtained by comparing the different cooking procedures at the same cooking time (data not shown), it is noteworthy to underline that the levels of citric acid and glucobrassicin drastically decreased after 10 min of boiling whereas they were not affected by 10 min of steaming or *sous-vide*; *p*-coumaric and gallic acids were stable in brassica vegetables cooked with the sous vide technique [46]. The level of glucosativin increased only after 10 min of steaming and sous vide. Furthermore, 25 min of boiling seems to produce an important decrease in most of the cases in comparison to the raw material, while for samples that were steamed or sous vide, similar concentration levels to those observed for the non-cooked samples were found. This is the case of 2-phenylethylglucosinolate (gluconasturtin), 1-sinapoyl-2-feruoyl-gentiobiose and quercitin-3-caffeoylsophorotrioside-7-glucoside and quercetin-3-O-sinapoyl-sophoroside-7-O-glucoside. In the case of high cooking times (40 min, no data were obtained for the boiling procedure), steaming and sous vide affected solely the level of the courmaroyl-diglucoside, which was significantly enhanced.

#### 3.3.2. Orange Cauliflower

Considering the same thermal treatment, the panel of phytochemicals found to be more discriminant for orange cauliflower was mostly different from that revealed for the violet one. These findings could be related to the differences in phytochemical composition between the cauliflowers.

In detail, boiling causes an important increase in caffeoyl-quinic acid and glucosativin levels and a decrease in kaempferol 7-O-glucoside. Sikora et al. [36] studied the effect of boiling on the content of flavonoids in white and green cauliflowers. Higher amounts of quercetin and kaempferol were found in fresh samples compared to the boiled ones.

Steaming treatment seems to produce only increases in the concentration levels of those phytochemicals found to be more discriminant among orange cauliflower samples, following two trends in the case of the violet cauliflower. For some compounds (i.e., kaempferol-3-O-feruloyl-sophoroside-7-O-glucoside, kaempferol-3-O-hydroxyferuloyl-sophoroside-7-O-glucoside, caffeoyl-quinic acid, and quercetin-3-O-sinapoyl-sophoroside) their levels are increasing with the steaming treatment time, while for salicyloyl-glucose, trisinapoylgentionbiose, and glucosativin, an increase is observed and then the concentration levels decrease when the samples are over-steamed.

Like steaming treatment, for those phytochemicals found to be discriminant among the sous vide orange cauliflower samples, two trends are observed. In general, concentration levels increased, such as for caffeoyl-quinic and sinapic acids, 4-mercaptobutyl, 4-(methylthio) butyl, and km 3-sinapoylsophorotrioside-7-glucoside, among others, or increased only at low sous vide treatment times, and then decreased for over-treated samples. This was the behavior observed for trisinapoylgentionbiose, sinapoyl-feruloyltentiobiose, salicyloyl-glucose, or sinapylglucoside.

However, when the same discriminant was revealed for both cauliflowers, the impact of thermal treatment on the concentration level of the compounds was identical. In fact, for both cauliflowers, the boiling decreased the sinapic acid level, steaming increased glucosativin, and sous vide enhanced the caffeoyl-quinic acid level. For the first time, glucosativin was found as the discriminant chemical descriptor in colored cauliflower submitted to cooking. Glucosativin is the main glucosinolate present in rocket and E. sativa and its product breakdown is the isothiocyanate sativin, responsible for health effects in the body and the aroma typical of rocket [47]. This compound could be related to the sensorial properties of cooked cauliflowers. In fact, glucosinolates are correlated to the sensorial acceptance of brassica, but this area of research is unexplored [35].

## 4. Conclusions

For the first time, a UHPLC–HRMS analysis of phytochemical profile (phenols and glucosinolates) combined with chemometrics was successfully employed as an efficient tool to differentiate cauliflowers according to steam and water cooking. Boiling showed a decrement in organic acids, glucobrassicin, and kaempferol-7-o-glucoside as chemical discriminants. Steaming and sous vide showed an increment in glucosativin, kaempferol-7-o-glucoside, some kaempferol derivates, and hydroxycinnamic acids derivates. For the first time, glucosativin was used as a chemical descriptor in sous vide and steamed cauliflowers. Koss-Mikołajczyk et al. stated that the matrix effect could be a key factor in cauliflower health-promoting activity [48]. We stressed that concept, highlighting a strong relationship between the impact of thermal treatment and the cauliflower variety.

In general, the findings could improve the knowledge of the relation of sensorial (e.g., sulfur odor from glucosinolates, bitterness, and astringency from polyphenols) and phytochemical profile of brassica vegetables, a new area of interest [35]. Our results could allow food companies to develop and deliver validated products from a nutritional point of view. Down the line, the selection of a pattern of markers could be quantified to discriminate food processed differently.

## Data Availability

The data used to support the findings of this study can be made available by the corresponding author upon request.

## Supplementary Materials

The following supporting information can be downloaded at: https://www.mdpi.com/article/10.3390/foods11193041/s1, Figure S1: PCA Score plot of PC1 vs. PC2 when UHPLC–HRMS phytochemical profiles were employed as chemical descriptors of all the orange cauliflower samples analyzed; Figure S2: Histograms (normalized peak area) of the level variation of the most discriminant phytochemicals (VIP) obtained for violet cauliflower according to the cooking procedure employed: (a) boiling, (b) steaming, (c) *sous*-*vide*; Table S1: TraceFinder TM Accurate mass database employed.

## References

[1] Aǧagündüz D., Şahin T.Ö., Yilmaz B., Ekenci K.D., Duyar Özer Ş., Capasso R. (2022). Cruciferous Vegetables and Their Bioactive Metabolites: From Prevention to Novel Therapies of Colorectal Cancer. Evid. Based. Complement. Alternat. Med..

[2] Mageney V., Neugart S., Albach D.C. (2017). A Guide to the Variability of Flavonoids in *Brassica oleracea*. Molecules.

[3] Aguilera Y., Herrera T., Benítez V., Arribas S.M., López De Pablo A.L., Esteban R.M., Martín-Cabrejas M.A. (2015). Estimation of Scavenging Capacity of Melatonin and Other Antioxidants: Contribution and Evaluation in Germinated Seeds. Food Chem..

[4] Zietz M., Weckmüller A., Schmidt S., Rohn S., Schreiner M., Krumbein A., Kroh L.W. (2010). Genotypic and Climatic Influence on the Antioxidant Activity of Flavonoids in Kale (*Brassica oleracea* Var. *Sabellica*). J. Agric. Food Chem..

[5] Oueslati S., Ellili A., Legault J., Pichette A., Ksouri R., Lachaal M., Karray-Bouraoui N. (2015). Phenolic Content, Antioxidant and Anti-Inflammatory Activities of Tunisian *Diplotaxis Simplex* (Brassicaceae). Nat. Prod. Res..

[6] Farhat G., Drummond S., Al-Dujaili E.A.S. (2017). Polyphenols and Their Role in Obesity Management: A Systematic Review of Randomized Clinical Trials. Phyther. Res..

[7] Guasch-Ferré M., Merino J., Sun Q., Fitó M., Salas-Salvadó J. (2017). Dietary Polyphenols, Mediterranean Diet, Prediabetes, and Type 2 Diabetes: A Narrative Review of the Evidence. Oxid. Med. Cell. Longev..

[8] Chiva-Blanch G., Badimon L. (2017). Effects of Polyphenol Intake on Metabolic Syndrome: Current Evidences from Human Trials. Oxid. Med. Cell. Longev..

[9] Hossen M.S., Ali M.Y., Jahurul M.H.A., Abdel-Daim M.M., Gan S.H., Khalil M.I. (2017). Beneficial Roles of Honey Polyphenols against Some Human Degenerative Diseases: A Review. Pharmacol. Rep..

[10] Mattioli R., Francioso A., D’Erme M., Trovato M., Mancini P., Piacentini L., Casale A.M., Wessjohann L., Gazzino R., Costantino P. (2019). Anti-Inflammatory Activity of a Polyphenolic Extract from Arabidopsis Thaliana in in Vitro and in Vivo Models of Alzheimer’s Disease. Int. J. Mol. Sci..

[11] Bahramsoltani R., Ebrahimi F., Farzaei M.H., Baratpourmoghaddam A., Ahmadi P., Rostamiasrabadi P., Rasouli Amirabadi A.H., Rahimi R. (2019). Dietary Polyphenols for Atherosclerosis: A Comprehensive Review and Future Perspectives. Crit. Rev. Food Sci. Nutr..

[12] Chen A.Y., Chen Y.C. (2013). A Review of the Dietary Flavonoid, Kaempferol on Human Health and Cancer Chemoprevention. Food Chem..

[13] Neugart S., Baldermann S., Hanschen F.S., Klopsch R., Wiesner-Reinhold M., Schreiner M. (2018). The Intrinsic Quality of Brassicaceous Vegetables: How Secondary Plant Metabolites Are Affected by Genetic, Environmental, and Agronomic Factors. Sci. Hortic..

[14] Russo G.L., Tedesco I., Spagnuolo C., Russo M. (2017). Antioxidant Polyphenols in Cancer Treatment: Friend, Foe or Foil?. Semin. Cancer Biol..

[15] Šamec D., Urlić B., Salopek-Sondi B. (2019). Kale (*Brassica oleracea* Var. *Acephala*) as a Superfood: Review of the Scientific Evidence behind the Statement. Crit. Rev. Food Sci. Nutr..

[16] Wu X., Huang H., Childs H., Wu Y., Yu L., Pehrsson P.R. (2021). Glucosinolates in *Brassica* Vegetables: Characterization and Factors That Influence Distribution, Content, and Intake. Annu. Rev. Food Sci. Technol..

[17] Mazumder A., Dwivedi A., Plessis J. (2016). Du Sinigrin and Its Therapeutic Benefits. Molecules.

[18] Abbaoui B., Lucas C.R., Riedl K.M., Clinton S.K., Mortazavi A. (2018). Cruciferous Vegetables, Isothiocyanates, and Bladder Cancer Prevention. Mol. Nutr. Food Res..

[19] Arumugam A., Razis A.F.A. (2018). Apoptosis as a Mechanism of the Cancer Chemopreventive Activity of Glucosinolates: A Review. Asian Pac. J. Cancer Prev..

[20] Avato P., Argentieri M.P. (2015). Brassicaceae: A Rich Source of Health Improving Phytochemicals. Phytochem. Rev..

[21] Fujioka N., Fritz V., Upadhyaya P., Kassie F., Hecht S.S. (2016). Research on Cruciferous Vegetables, Indole-3-Carbinol, and Cancer Prevention: A Tribute to Lee W. Wattenberg. Mol. Nutr. Food Res..

[22] Kumar G., Tuli H.S., Mittal S., Shandilya J.K., Tiwari A., Sandhu S.S. (2015). Isothiocyanates: A Class of Bioactive Metabolites with Chemopreventive Potential. Tumor Biol..

[23] Herz C., Márton M.R., Tran H.T.T., Gründemann C., Schell J., Lamy E. (2016). Benzyl Isothiocyanate but Not Benzyl Nitrile from Brassicales Plants Dually Blocks the COX and LOX Pathway in Primary Human Immune Cells. J. Funct. Foods.

[24] Guzmán-Pérez V., Bumke-Vogt C., Schreiner M., Mewis I., Borchert A., Pfeiffer A.F.H. (2016). BenzylglucosinolateDerived Isothiocyanate from Tropaeolum Majus Reduces Gluconeogenic Gene and Protein Expression in Human Cells. PLoS ONE.

[25] Waterman C., Rojas-Silva P., Tumer T.B., Kuhn P., Richard A.J., Wicks S., Stephens J.M., Wang Z., Mynatt R., Cefalu W. (2015). Isothiocyanate-Rich *Moringa Oleifera* Extract Reduces Weight Gain, Insulin Resistance, and Hepatic Gluconeogenesis in Mice. Mol. Nutr. Food Res..

[26] Orlando P., Nartea A., Silvestri S., Marcheggiani F., Cirilli I., Dludla P.V., Fiorini R., Pacetti D., Loizzo M.R., Lucci P. (2022). Bioavailability Study of Isothiocyanates and Other Bioactive Compounds of *Brassica oleracea* L. Var. *Italica* Boiled or Steamed: Functional Food or Dietary Supplement?. Antioxidants.

[27] Thomas M., Badr A., Desjardins Y., Gosselin A., Angers P. (2018). Characterization of Industrial Broccoli Discards (*Brassica oleracea* Var. *Italica*) for Their Glucosinolate, Polyphenol and Flavonoid Contents Using UPLC MS/MS and Spectrophotometric Methods. Food Chem..

[28] Saavedra-Leos M.Z., Leyva-Porras C., Toxqui-Terán A., Espinosa-Solis V. (2021). Physicochemical Properties and Antioxidant Activity of Spray-Dry Broccoli (*Brassica oleracea* Var. *Italica*) Stalk and Floret Juice Powders. Molecules.

[29] Ramirez D., Abellán-Victorio A., Beretta V., Camargo A., Moreno D.A. (2020). Functional Ingredients from Brassicaceae Species: Overview and Perspectives. Int. J. Mol. Sci..

[30] Kapusta-Duch J., Szeląg-Sikora A., Sikora J., Niemiec M., Gródek-Szostak Z., Kuboń M., Leszczyńska T., Borczak B. (2019). Health-Promoting Properties of Fresh and Processed Purple Cauliflower. Sustainability.

[31] Nartea A., Fanesi B., Falcone P.M., Pacetti D., Frega N.G., Lucci P. (2021). Impact of Mild Oven Cooking Treatments on Carotenoids and Tocopherols of Cheddar and Depurple Cauliflower (*Brassica oleracea* L. Var. *Botrytis*). Antioxidants.

[32] Nartea A., Falcone P.M., Torri L., Ghanbarzadeh B., Frega N.G., Pacetti D. (2021). Modeling Softening Kinetics at Cellular Scale and Phytochemicals Extractability in Cauliflower under Different Cooking Treatments. Foods.

[33] Florkiewicz A., Ciska E., Filipiak-Florkiewicz A., Topolska K. (2017). Comparison of Sous-Vide Methods and Traditional Hydrothermal Treatment on GLS Content in *Brassica* Vegetables. Eur. Food Res. Technol..

[34] Kapusta-Duch J., Kusznierewicz B., Leszczyńska T., Borczak B. (2016). Effect of Cooking on the Contents of Glucosinolates and Their Degradation Products in Selected *Brassica* Vegetables. J. Funct. Foods.

[35] Wieczorek M.N., Dunkel A., Szwengiel A., Czaczyk K., Drożdżyńska A., Zawirska-Wojtasiak R., Jeleń H.H. (2022). The Relation between Phytochemical Composition and Sensory Traits of Selected *Brassica* Vegetables. LWT.

[36] Martínez S., Armesto J., Gómez-Limia L., Carballo J. (2020). Impact of Processing and Storage on the Nutritional and Sensory Properties and Bioactive Components of *Brassica* Spp. A Review. Food Chem..

[37] Dos Reis L.C.R., de Oliveira V.R., Hagen M.E.K., Jablonski A., Flores S.H., de Oliveira Rios A. (2015). Effect of Cooking on the Concentration of Bioactive Compounds in Broccoli (*Brassica oleracea* Var. *Alphina F1*) Grown in an Organic System. Food Chem..

[38] Mazzeo T., N’Dri D., Chiavaro E., Visconti A., Fogliano V., Pellegrini N. (2011). Effect of Two Cooking Procedures on Phytochemical Compounds, Total Antioxidant Capacity and Colour of Selected Frozen Vegetables. Food Chem..

[39] Wachtel-Galor S., Wong K.W., Benzie I.F.F. (2008). The Effect of Cooking on *Brassica* Vegetables. Food Chem..

[40] Pellegrini N., Chiavaro E., Gardana C., Mazzeo T., Contino D., Gallo M., Riso P., Fogliano V., Porrini M. (2010). Effect of Different Cooking Methods on Color, Phytochemical Concentration, and Antioxidant Capacity of Raw and Frozen *Brassica* Vegetables. J. Agric. Food Chem..

[41] Bongoni R., Verkerk R., Steenbekkers B., Dekker M., Stieger M. (2014). Evaluation of Different Cooking Conditions on Broccoli (*Brassica oleracea* Var. *Italica*) to Improve the Nutritional Value and Consumer Acceptance. Plant Foods Hum. Nutr..

[42] Barbosa S., Pardo-Mates N., Hidalgo-Serrano M., Saurina J., Puignou L., Núnez O. (2018). Detection and Quantitation of Frauds in the Authentication of Cranberry-Based Extracts by UHPLC-HRMS (Orbitrap) Polyphenolic Profiling and Multivariate Calibration Methods. J. Agric. Food Chem..

[43] Massart D.L., Vandeginste B.G.M., Buydens L.M.C., de Jong S., Lewi P.J., Smeyers-Verbeke J. (1997). Handbook of Chemometrics and Qualimetrics.

[44] Rothwell J.A., Medina-Remón A., Pérez-Jiménez J., Neveu V., Knaze V., Slimani N., Scalbert A. (2015). Effects of Food Processing on Polyphenol Contents: A Systematic Analysis Using Phenol-Explorer Data. Mol. Nutr. Food Res..

[45] Wu X., Zhao Y., Haytowitz D.B., Chen P., Pehrsson P.R. (2019). Effects of Domestic Cooking on Flavonoids in Broccoli and Calculation of Retention Factors. Heliyon.

[46] Florkiewicz A., Socha R., Filipiak-Florkiewicz A., Topolska K. (2019). Sous-Vide Technique as an Alternative to Traditional Cooking Methods in the Context of Antioxidant Properties of *Brassica* Vegetables. J. Sci. Food Agric..

[47] Fechner J., Kaufmann M., Herz C., Eisenschmidt D., Lamy E., Kroh L.W., Hanschen F.S. (2018). The Major Glucosinolate Hydrolysis Product in Rocket (*Eruca sativa* L.), Sativin, Is 1,3-Thiazepane-2-Thione: Elucidation of Structure, Bioactivity, and Stability Compared to Other Rocket Isothiocyanates. Food Chem..

[48] Koss-Mikołajczyk I., Kusznierewicz B., Wiczkowski W., Płatosz N., Bartoszek A. (2019). Phytochemical Composition and Biological Activities of Differently Pigmented Cabbage (*Brassica oleracea* Var. *Capitata*) and Cauliflower (*Brassica oleracea* Var. *Botrytis*) Varieties. J. Sci. Food Agric..

