# Supplementary material for: Glucosinolates and Polyphenols of Colored Cauliflower as Chemical Discriminants Based on Cooking Procedures
Source: Foods. 2022 Sep 30;11(19):3041. doi: 10.3390/foods11193041 (PMC9563729; doi:10.3390/foods11193041)
Supplement: Supplementary file 1 [file foods-11-03041-s001.zip › foods-1875339-supplementary.pdf]

SUPLEMENTARY MATERIAL

Impact of Cooking Methods in Phytochemical Profile of Violet and Orange Cauliflower (*Brassica Oleracea* L. var. *botrytis*) by UHPLC-HRMS (Orbitrap).

Ancuta Nartea [1], Benedetta Fanesi [1], Alessandra Giardinieri [1], Guillem Campmajó [2,3], Paolo Lucci [1], Javier Saurina [2,3], Deborah Pacetti [1,*], Dennis Fiorini [4], Natale Giuseppe Frega [1] and Oscar Núñez [2,3]

[1] Department of Agricultural, Food and Environmental Sciences, Marche Polytechnic University, Via Brecce Bianche, 60131 Ancona, Italy
[2] Department of Chemical Engineering and Analytical Chemistry, University of Barcelona, Martí i Franquès 1-11, E-08028 Barcelona, Spain
[3] Research Institute in Food Nutrition and Food Safety, University of Barcelona, Av. Prat de la Riba 171, Edifici Recerca (Gaudí), E-08901 Santa Coloma de Gramenet, Barcelona, Spain
[4] School of Science and Technology, Chemistry Division, University of Camerino, V. S. Agostino 1, Camerino, 62032 Macerata, Italy
* Correspondence: d.pacetti@univpm.it

Table S1. TraceFinder™ Accurate mass database employed.

**Tentative identification**	**Chemical Formula**	**Accurate mass m/z [M-H]⁻**
**ORGANIC ACIDS**		
Malic acid	$C_4H_6O_5$	133.0142
Citric acid	$C_6H_8O_7$	191.0197
Methylcitric acid	$C_7H_{10}O_7$	205.0353
**PHENOLIC COMPOUNDS**		
Hydroxybenzoic acids		
Protocatechuic acid	$C_7H_6O_4$	153.0193
Hydroxycinnamic acids derivatives		
Ferulic acid	$C_{10}H_{10}O_4$	193.0506
Sinapic acid	$C_{11}H_{12}O_5$	223.0612
Salicyloyl-glucose	$C_{13}H_{16}O_8$	299.0772
Caffeoyl-quinic acid	$C_{16}H_{18}O_9$	353.0878
Feruloylglucoside	$C_{16}H_{20}O_9$	355.1034
Sinapylglucoside	$C_{17}H_{22}O_{10}$	385.1140
Courmaroyl-diglucoside	$C_{21}H_{28}O_{14}$	503.1406
Sinapoyl-gentiobiose	$C_{23}H_{32}O_{15}$	547.1668
1-Sinapoyl-2-feruloyl-gentiobiose	$C_{33}H_{40}O_{18}$	723.2141
1,2-disinapoylgentiobiose	$C_{34}H_{42}O_{19}$	753.2247
1,2′-disinapoyl-2-feruloylgentiobiose	$C_{44}H_{50}O_{22}$	929.2721
1,2,2′-trisinapoylgentiobiose	$C_{45}H_{52}O_{23}$	959.2826
Isorhamnetin derivatives		
Isorhamnetin-3,7-*O*-diglucoside, Isorhamnetin-3-*O*-diglucoside	$C_{28}H_{32}O_{17}$	639.1566
Isorhamnetin-3-*O*-sinapoylglucoside-7-*O*-glucoside	$C_{39}H_{42}O_{21}$	845.2145
Kaempferol derivatives		
Kaempferol-7-*O*-glucoside	$C_{21}H_{20}O_{11}$	447.0932

Kaempferol-3-*O*-glucoside-7-*O*-rhanmoside	$C_{27}H_{30}O_{15}$	593.1511
Kaempferol-3,7-di-*O*-glucoside, Kaempferol-3-*O*-diglucoside	$C_{27}H_{30}O_{16}$	609.1461
Kaempferol-3-*O*-sophoroside-7-*O*-glucoside	$C_{33}H_{40}O_{21}$	771.1989
Kaempferol-3-*O*-*p*-coumaroyl-sophoroside-7-*O*-glucoside	$C_{42}H_{46}O_{23}$	917.2357
Kaempferol-3-O-sophorotrioside-7-glucoside<br>Kaempferol-3-caffeoyldiglucoside-7-glucoside	$C_{42}H_{46}O_{24}$	933.2306
Kaempferol 7-*O*-glucoside-3-O-acyl glucosyls (Kaempferol tretraglucoside)	$C_{42}H_{48}O_{24}$	935.2462
Kaempferol-3-*O*-feruloyl-sophoroside-7-*O*-glucoside	$C_{43}H_{48}O_{24}$	947.2462
Kaempferol-3-*O*-sophoroside-7-*O*-sinapoyl-rhamnoside	$C_{44}H_{50}O_{24}$	961.2619
Kaempferol-3-*O*-hydroxyferuloyl-sophoroside-7-*O*-glucoside	$C_{43}H_{48}O_{25}$	963.2411
Kaempferol-3-*O*-sinapoyl-sophoroside-7-*O*-glucoside<br>Quercetin-3-*O*-sophoroside-7-*O*-sinapoyl-rhamoside	$C_{44}H_{50}O_{25}$	977.2568
Kaempferol-3-*O*-feruloyl-sophorotrioside-7-*O*-glucoside	$C_{49}H_{58}O_{29}$	1109.2991
Kaempferol-3-*O*-*p*-coumaroyl-sophoroside-7-*O*-diglucoside	$C_{48}H_{56}O_{28}$	1079.2885
Kaempferol-3-*O*-caffeoyl-sophorotrioside-7-*O*-glucoside<br>Kaempferol-3-*O*-caffeoyl-triglucoside-7-*O*-glucoside<br>Quercetin-3-*O*-*p*-coumaroyl-triglucoside-7-*O*-glucoside	$C_{48}H_{56}O_{29}$	1095.2834
Kaempferol-3-*O*-hydroxyferuloyl-sophorotrioside-7-*O*-glucoside	$C_{49}H_{58}O_{30}$	1125.2940
Kaempferol-3-*O*-sinapoyl-sophorotrioside-7-*O*-glucoside	$C_{50}H_{60}O_{30}$	1139.3096
Kaempferol-3-*O*-sinapoyl-feruloyl-sophoroside-7-*O*-glucoside	$C_{54}H_{58}O_{28}$	1153.3041
Kaempferol-3-sinapoyl-diglucoside-7-sinapoylglucoside<br>Kaempferol-3-disinapoyl-diglucoside-7-glucoside	$C_{55}H_{60}O_{29}$	1183.3147
Quercetin derivatives		
Quercetin-3-*O*-diglucoside, Quercetin-7-sophoroside	$C_{27}H_{30}O_{17}$	625.1410
Quercetin-3-*O*-sophoroside-7-*O*-glucoside	$C_{33}H_{40}O_{22}$	787.1938
Quercetin-3-*O*-sinapoyl-sophoroside	$C_{38}H_{40}O_{21}$	831.1989
Quercetin-3-*O*-caffeoyl-sophoroside-7-*O*-glucoside	$C_{42}H_{46}O_{25}$	949.2255
Quercetin-3-*O*-hydroxyferuloyl-sophoroside-7-*O*-glucoside	$C_{43}H_{48}O_{26}$	979.2361
Quercetin-3-*O*-sinapoyl-diglucoside-7-*O*-glucoside	$C_{44}H_{50}O_{26}$	993.2517

Quercetin-3-*O*-sinapoyl-sophoroside-7-*O*-glucoside<br>Quercetin-3-*O*-sinapoyl-sophorotrioside		
Quercetin-3-*O*-caffeoyl-sophorotrioside-7-*O*-glucoside	$C_{48}H_{56}O_{30}$	1111.2783
Quercetin-3-*O*-hydroxyferuloyl-sophorotioside-7-*O*-glucoside	$C_{49}H_{58}O_{31}$	1141.2889
Quercetin-3-*O*-sinapoyl-triglucoside-7-*O*-glucoside	$C_{50}H_{60}O_{31}$	1155.3045
Quercetin-3-*O*-disinapoyl-sophorotrioside	$C_{55}H_{60}O_{30}$	1199.3096
**GLUCOSINOLATES**		
Aliphatic		
3-butenyl-glucosinolate (Gluconapin)	$C_{11}H_{19}NO_9S_2$	372.0428
4-pentenyl-glucosinolate (Glucobrassicanapin)	$C_{12}H_{21}NO_9S_2$	386.0585
(R)-2-hydroxy-3-butenylglucosinolate (Epi-progoitrin)	$C_{11}H_{19}NO_{10}S_2$	388.0377
2-propenylglucosinolate (Sinigrin)	$C_{10}H_{16}KNO_9S_2$	395.9830
4-mercaptobutylglucosinolate (Glucosativin)	$C_{11}H_{21}NO_9S_3$	406.0305
4-methylthiobutylglucosinolate (Glucoerucin)	$C_{12}H_{23}NO_9S_3$	420.0462
3-methylsulphinylpropylglucosinolate (Glucoiberin)	$C_{11}H_{21}NO_{10}S_3$	422.0254
4-methylthio-3-butenylglucosinolate (Dehydroerucin)	$C_{12}H_{21}NO_{10}S_3$	434.0254
4-methylsulphinylbutylglucosinolate (Glucoraphanin)	$C_{12}H_{23}NO_{10}S_3$	436.0411
Indolic		
3-indolylmethylglucosinolate (Glucobrassicin)	$C_{16}H_{20}N_2O_9S_2$	447.0537
4-hydroxy-3-indolylmethylglucosinolate (4-Hydroxyglucobrassicin)	$C_{16}H_{20}N_2O_{10}S_2$	463.0486
1-methoxy-3-indolylmethylglucosinolate (Neoglucobrassicin)<br>4-methoxy-3-indolylmethylglucosinolate (4-Methoxyglucobrassicin)	$C_{17}H_{22}N_2O_{10}S_2$	477.0643
Aromatic		
Benzyl-glucosinolate (Glucotropaeolin)	$C_{14}H_{19}NO_9S_2$	408.0428
2-phenylethyl-glucosinolate (Gluconasturtiin)	$C_{15}H_{21}NO_9S_2$	422.0585

Figure S1. PCA Score plot of PC1 vs PC2 when UHPLC-HRMS phytochemical profiles where employed as chemical descriptors of all the orange and violet cauliflower samples analyzed.

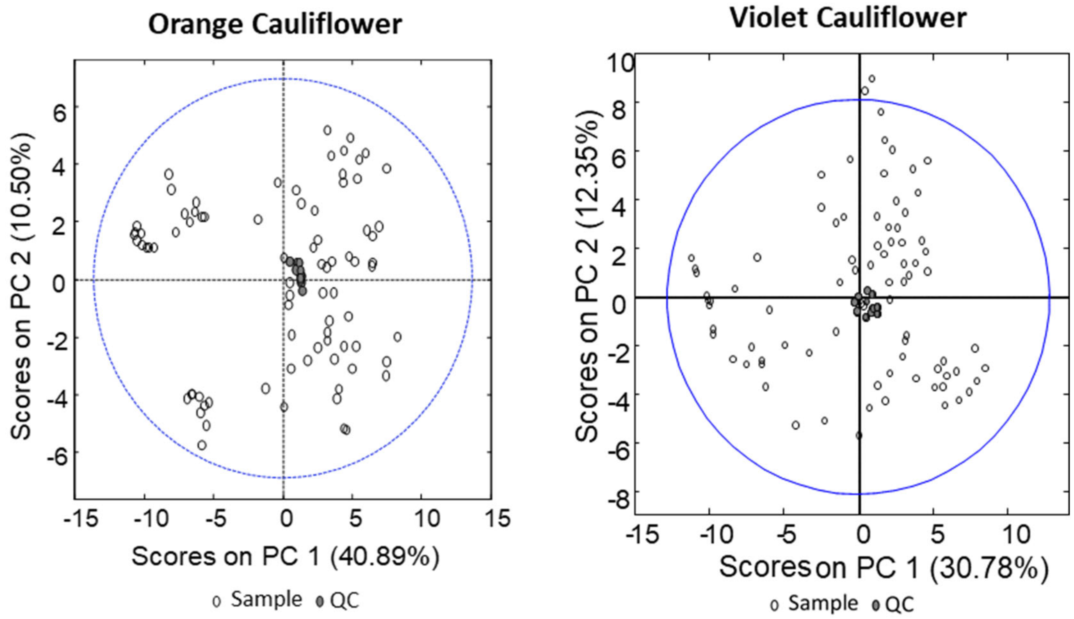

Figure S2. Scatterplots (normalized peak area) of the level variation of the most discriminant phytochemicals (VIP) obtained for violet cauliflower according to the cooking procedure employed. Black lines show the medians and open cycles the replicates.

a) Boiling

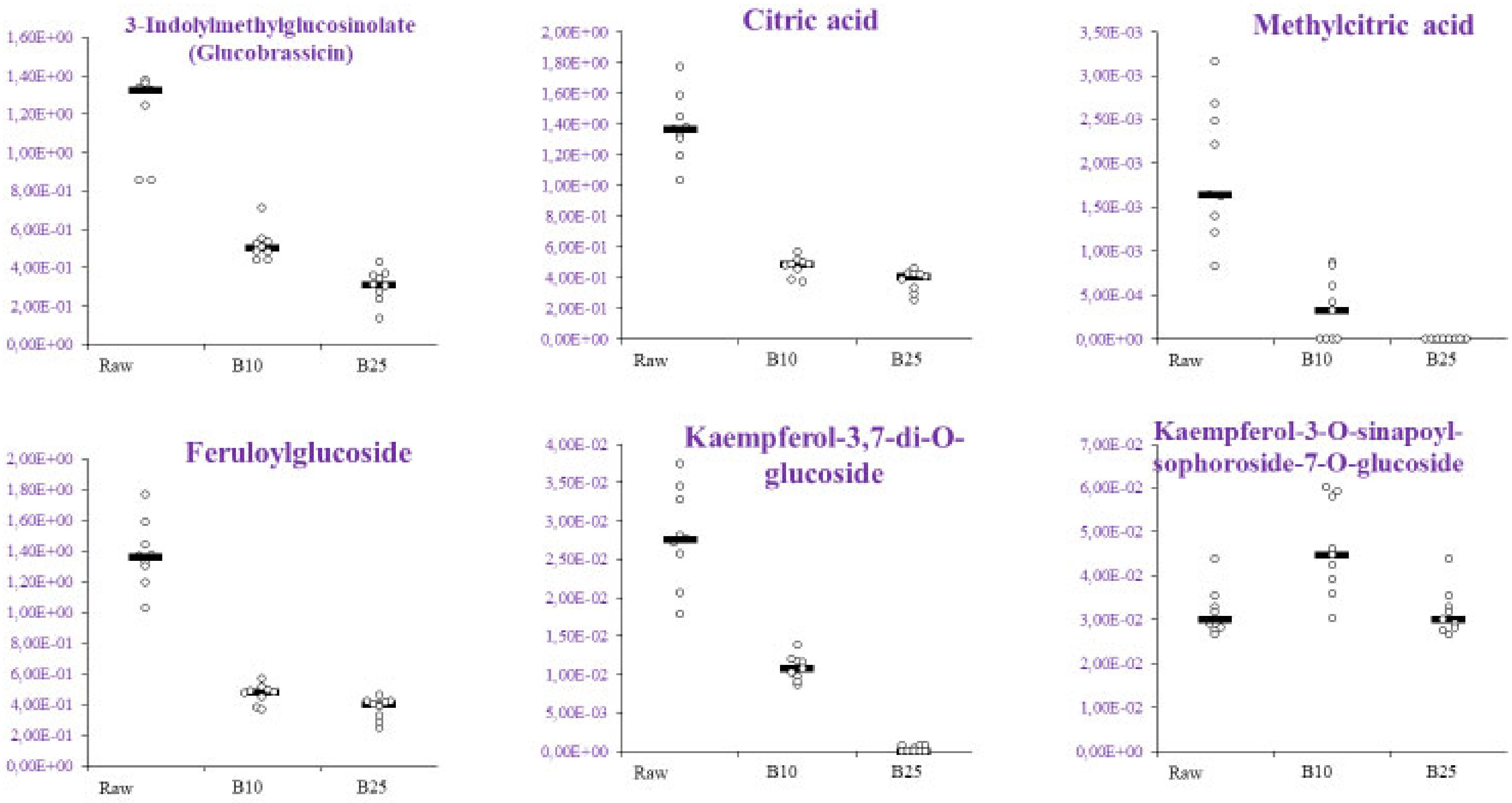

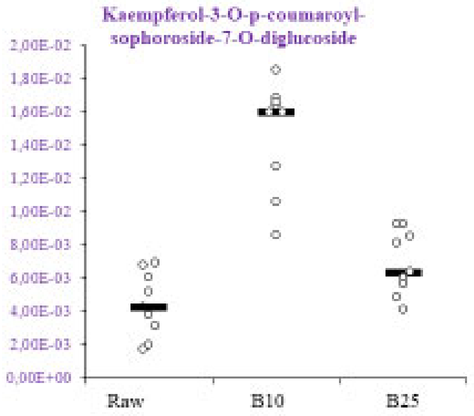
Kaempferol-3-O-p-coumaroyl-sophoroside-7-O-diglucoside
2,00E-02
1,80E-02
1,60E-02
1,40E-02
1,20E-02
1,00E-02
8,00E-03
6,00E-03
4,00E-03
2,00E-03
0,00E+00
Raw
B10
B25

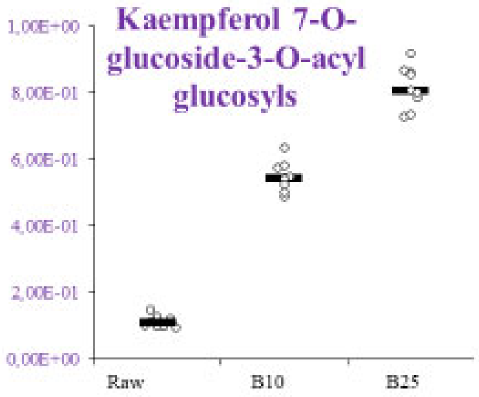
Kaempferol 7-O-glucoside-3-O-acyl glucosyls
1,00E+00
8,00E-01
6,00E-01
4,00E-01
2,00E-01
0,00E+00
Raw
B10
B25

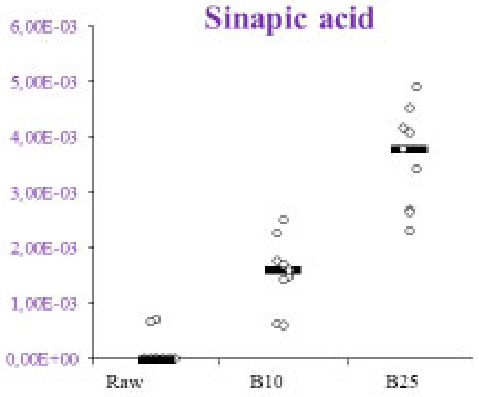
Sinapic acid
6,00E-03
5,00E-03
4,00E-03
3,00E-03
2,00E-03
1,00E-03
0,00E+00
Raw
B10
B25

b) Steaming

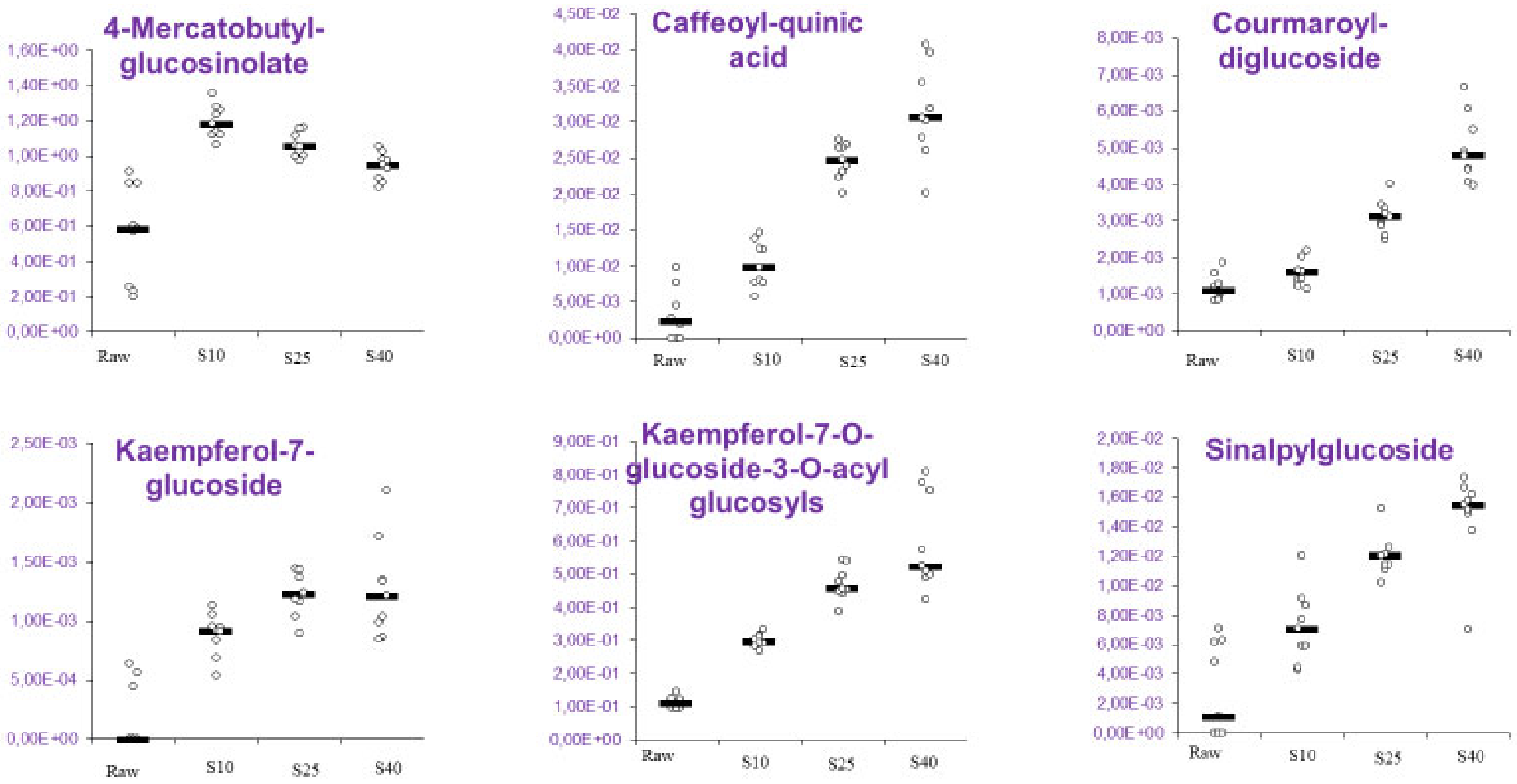

4-Mercatobutyl-glucosinolate
1,60E+00
1,40E+00
1,20E+00
1,00E+00
8,00E-01
6,00E-01
4,00E-01
2,00E-01
0,00E+00
Raw
S10
S25
S40
Caffeoyl-quinic acid
4,50E-02
4,00E-02
3,50E-02
3,00E-02
2,50E-02
2,00E-02
1,50E-02
1,00E-02
5,00E-03
0,00E+00
Raw
S10
S25
S40
Courmaroyl-diglucoside
8,00E-03
7,00E-03
6,00E-03
5,00E-03
4,00E-03
3,00E-03
2,00E-03
1,00E-03
0,00E+00
Raw
S10
S25
S40
Kaempferol-7-glucoside
2,50E-03
2,00E-03
1,50E-03
1,00E-03
5,00E-04
0,00E+00
Raw
S10
S25
S40
Kaempferol-7-O-glucoside-3-O-acyl glucosyls
9,00E-01
8,00E-01
7,00E-01
6,00E-01
5,00E-01
4,00E-01
3,00E-01
2,00E-01
1,00E-01
0,00E+00
Raw
S10
S25
S40
Sinalpylglucoside
2,00E-02
1,80E-02
1,60E-02
1,40E-02
1,20E-02
1,00E-02
8,00E-03
6,00E-03
4,00E-03
2,00E-03
0,00E+00
Raw
S10
S25
S40

c) Sous-vide

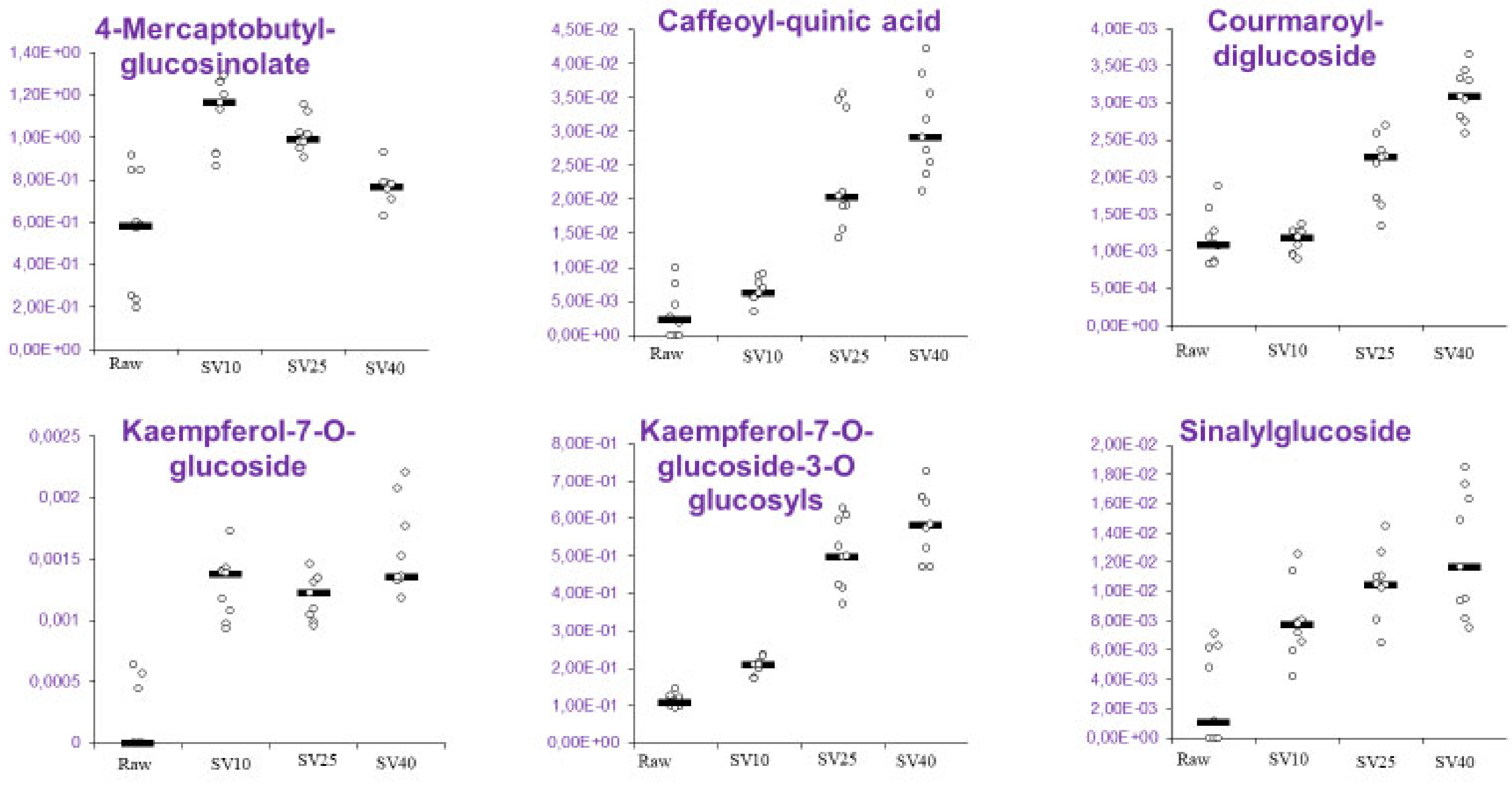

4-Mercaptobutyl-glucosinolate
1,40E+00
1,20E+00
1,00E+00
8,00E-01
6,00E-01
4,00E-01
2,00E-01
0,00E+00
Raw
SV10
SV25
SV40
Caffeoyl-quinic acid
4,50E-02
4,00E-02
3,50E-02
3,00E-02
2,50E-02
2,00E-02
1,50E-02
1,00E-02
5,00E-03
0,00E+00
Raw
SV10
SV25
SV40
Courmaroyl-diglucoside
4,00E-03
3,50E-03
3,00E-03
2,50E-03
2,00E-03
1,50E-03
1,00E-03
5,00E-04
0,00E+00
Raw
SV10
SV25
SV40
Kaempferol-7-O-glucoside
0,0025
0,002
0,0015
0,001
0,0005
0
Raw
SV10
SV25
SV40
Kaempferol-7-O-glucoside-3-O glucosyls
8,00E-01
7,00E-01
6,00E-01
5,00E-01
4,00E-01
3,00E-01
2,00E-01
1,00E-01
0,00E+00
Raw
SV10
SV25
SV40
Sinalylglucoside
2,00E-02
1,80E-02
1,60E-02
1,40E-02
1,20E-02
1,00E-02
8,00E-03
6,00E-03
4,00E-03
2,00E-03
0,00E+00
Raw
SV10
SV25
SV40
